# Development and refinement of the open dialog adherence protocol in complex mental health care

**DOI:** 10.3389/fpsyg.2022.1041375

**Published:** 2023-01-06

**Authors:** Melissa Lotmore, Douglas Ziedonis, Mauricio Alvarez Monjaras, Mark Hopfenbeck, Russell Razzaque, Emily Wilson, Stephen Pilling

**Affiliations:** ^1^Department of Clinical, Educational and Health Psychology, University College London, London, United Kingdom; ^2^Department of Health Sciences, The University of New Mexico, Albuquerque, NM, United States; ^3^Department of Health Sciences, Norwegian University of Science and Technology, Trondheim, Sør-Trøndelag, Norway; ^4^North East London NHS Foundation Trust, London, United Kingdom; ^5^Camden and Islington NHS Foundation Trust, London, United Kingdom

**Keywords:** open dialogue, mental health, adherence, reliability, protocol, measure

## Abstract

**Introduction:**

Open dialog (OD) is a both a therapeutic practice and a service delivery model that offers an integrated response to mental health care through mobilizing resources within the service user’s family and community networks through joint network meetings. Therapist adherence is a crucial to the effective delivery of interventions. A key way to measure this is through structured observation tools.

**Aims:**

The aim of this research project is to develop and refine the Dialogic Practice Adherence Scale, for use in OD research trials in the United Kingdom.

**Methods:**

This study was a mixed methods approach to the development of an OD practitioner adherence measure. Initial steps involved meetings and discussions with experts and a review of the literature. Content validation studies were completed using a modified Delphi technique. To assess reliability of the measure, OD network meetings were audio-recorded, and tapes were rated by two independent researchers. Inter-rater reliability and internal consistency were assessed through quantitative approaches assessing variance.

**Results:**

Results provide a description of how the OD Adherence Manual was developed in collaboration. Validation surveys showed high levels on consensus among experts in the field on the key elements of OD network meetings. Inter-rater reliability for the total score was excellent and internal consistency analyses suggest the scale is highly reliable.

**Discussion:**

The scale presented here is an initial attempt at rating practitioner adherence in OD network meetings. It provides encouraging evidence that this can be done with strong validity and reliability and can be completed by a range of raters with varying levels of clinical experience.

## 1. Introduction

At present in England, there is excessive pressure on psychiatric inpatient beds attributed to increased demand. This takes place in the context of reduced community resources, limitations in crisis response and decreasing availability of long-term community support (Wheeler et al., 2015). Individuals suffering from complex mental disorders, defined as emotional, cognitive, or behavioral disturbances that have reached a threshold that causes substantial functional impairment are most likely to be occupying these beds (Leichsenring and Rabung, 2008; Public Health England, 2018). These disorders have a long-term impact on the individual diagnosed and their support network and often require extensive interventions and multidisciplinary or multiagency team working (Horn, 1965; Keene, 2008).

Interventions that target the social network may have a role in ameliorating mental health crises, reducing the likelihood of relapse and therefore, help to decrease pressure on inpatient psychiatric beds (Hoult et al., 1983; Olivares et al., 2013). Although Community Recovery Home Treatment Teams (CRHTTs) often acknowledge and, may attempt to work with the social network of the person in crisis, the often-limited nature of CRHTT contact and poor coordination of services militates against this. Despite the early promise shown in randomized control trials (RCTs; Johnson et al., 2005) research suggests that CRHTTs may no longer be associated with a reduction in hospital admissions (Jacobs and Barrenho, 2011). This could be due to a considerable atrophy of the key functions of CRHTT with many services offering limited home visits outside of office hours and only 50% of services providing post-hospital discharge care (Wheeler et al., 2015).

Current service responses to these problems include the development of alternatives to admission (e.g., Crisis Houses; Lloyd-Evans et al., 2014), increased capacity for psychiatric assessment in Emergency Departments, and research aimed at improving CRHTT functioning [e.g., CORE program grant led by Johnson (2013)].^1^ However, these initiatives focus primarily on the management of the crisis and its aftermath, not the wider system change (e.g., continuing community support) which needs to be addressed if bed pressures are to be reduced and outcomes for service users improved in the longer term.

Epidemiological research implicates poor social networks in both the development and maintenance of mental disorder (Giacco et al., 2012). Interventions which target the social network have been advocated by developers of crisis services (e.g., Hoult in London in the 2000s) but given the brief nature of CRHTT contacts, limited staff knowledge and skills, and lack of continuity of care, such interventions are not currently provided. In addition, the evidence describing the content of these interventions, and how services which deliver them may be provided by the NHS is limited. One such model which may provide an alternative approach to crisis care is open dialog (OD). This approach explicitly focuses on bringing about change in the social network while supporting an individual through a mental health crisis. In depth exploration of the content of this approach is required for its potential implementation into the NHS.

Developed in Finland, OD is a both a therapeutic practice and a service delivery model. It offers an integrated response to mental health care with an emphasis on mobilizing resources within the service user’s family and community networks through joint network meetings (Seikkula et al., 2006, 2011). Network meetings are the core therapeutic intervention within the OD approach and often take place in service users own homes. In these network meetings, service users and their networks engage in shared decision making with professionals to deploy appropriate interventions (psychological, pharmaceutical, and/or social) with the aim of developing longer term mutual support. The development of an integrated OD approach to the provision of mental health services offers the possibility of an effective alternative to the current functional model where particular functions (e.g., crisis interventions, longer–term community support) are provided by separate teams.

A systematic review by Freeman et al. (2019) found 23 studies of OD (mixed methods, qualitative, and quantitative). The review suggests that although findings of these studies have been promising the evidence is low quality and RCTs are needed to draw any additional conclusions. Uncontrolled studies report reductions in bed usage and improved recovery rates following OD interventions (Seikkula et al., 2011). Although promising, there is no high-quality evidence to support an NHS-wide adoption of this model. In order to determine whether OD is an effective alternative to the current model, the ODDESSI program grant will undertake a multisite randomized control trial (RCT) comparing OD with treatment as usual (TAU). Findings from this RCT will influence whether or not changes are made more globally to NHS service structure to include more social network approaches. An important part of this research involves understanding what takes place in OD network meetings and how this links to therapeutic change.

**Figure 1:**
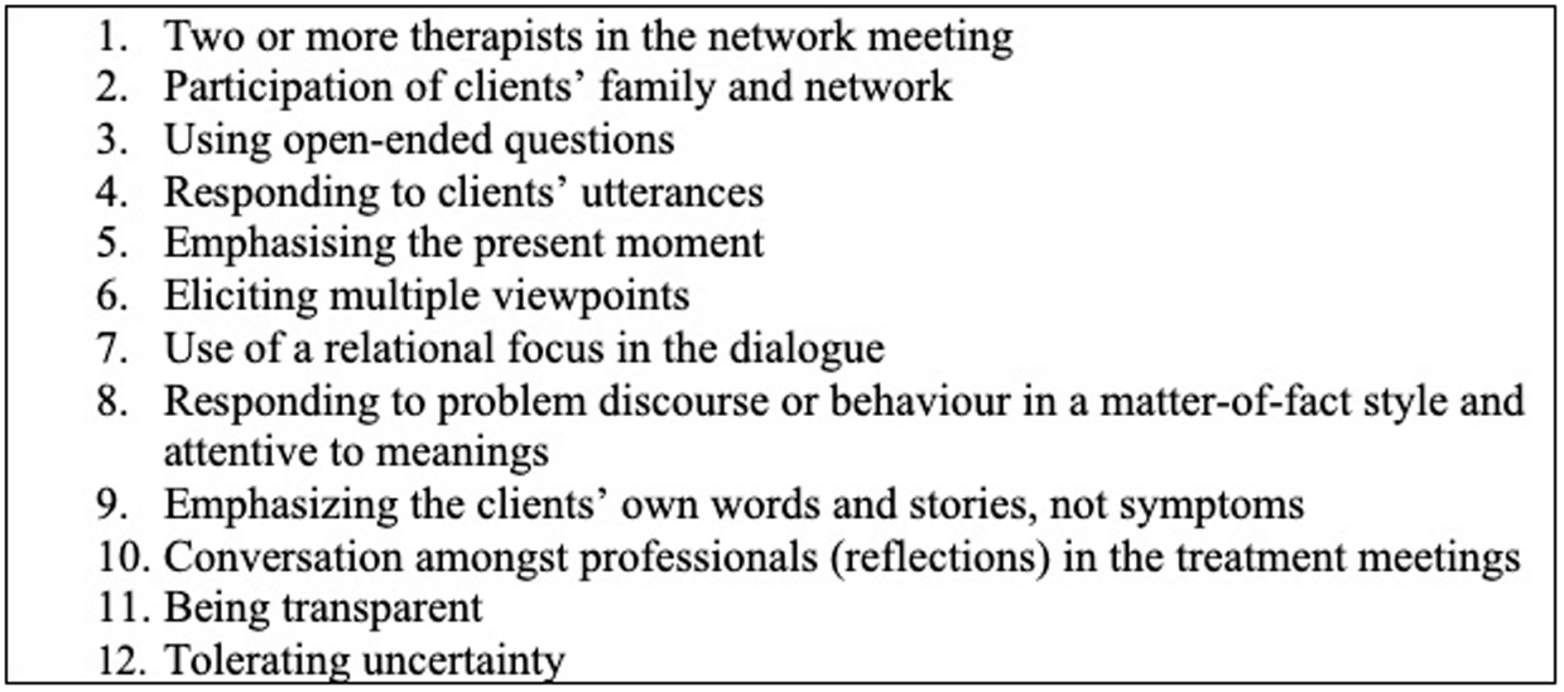
Key elements of dialogic paractice (Olson et al., 2014).

The central component of an OD network meeting is a dialogic interaction, in which the basic feature is that each participant feels heard and responded to. Being an OD practitioner involves being able to listen and adapt to the particular context and language of every exchange and it is not possible to make specific recommendations for sessions in advance (Olson et al., 2014). However, there are distinct elements on the part of the therapists that generate the flow of dialog which in turn helps to mobilize the resources of the person at the center of the network (Olson et al., 2014). As set out in *The Key Elements of Dialogic Practice in OD* (Olson et al., 2014), there are 12 key elements or “fidelity criteria” of dialogic practice which are important for understanding the OD model (presented in Figure 1). These elements describe ways in which the practitioners can use utterances to generate new narratives amongst network members and move away from problem saturated interactions.

In order to ensure adequate implementation of the OD model, measures of treatment integrity such as adherence and fidelity are required. These measures will provide information to researchers and treating teams about whether or not the OD approach is being delivered as developed and intended. This is necessary to link treatment to outcome which is the wider goal of the ODDESSI RCT. The Key Elements listed above may be a useful starting point for the development of a measure of practitioner adherence within OD network meetings as they have been identified by experts in the field as integral to the OD therapeutic process.

Therapist adherence is a crucial to the effective delivery of interventions, as well as necessary to support successful dissemination across settings (Startup et al., 2002; Lange et al., 2016). It is used to reflect the degree to which therapists employ interventions prescribed by a model or framework and avoid the use of proscribed interventions during their therapeutic exchanges with service-users (Yeaton and Sechrest, 1981; Waltz et al., 1993; Schoenwald et al., 2000). The principal way that adherence is measured is through structured observation scales – measures containing the key components of a model based on its theoretical constructs. These measures must be psychometrically robust in order to accurately measure adherence and be useful for ongoing research into the efficacy of an intervention (Glasgow et al., 2005; Gearing et al., 2011). Using these measures, treatment adherence research can provide information about the successes and failures in the delivery of a model linking symptom change with therapeutic progression based on specific intervention techniques (Startup and Shapiro, 1993; Hogue et al., 1998; Onwumere et al., 2009).

Adherence scales for OD have yet to be formally developed and tested (described below). They are required for use in the ODDESSI RCT to ensure accurate implementation of the model. A measure of practitioner adherence using the key elements described above will allow researchers to more clearly establish the content of OD network meetings, ensure its successful implementation, and link the therapeutic approach with outcomes.

The “Dialogic Practice Adherence Scale” (DPAS; Olson et al., n.d.), has been developed in the United States for their healthcare system based on expert knowledge and consensus. It is in its introductory phases and included only the 12 Key Elements and a rating scale. At present, it has not been evaluated, validated, nor has the measure been used in research trials which would subject it to rigorous reliability and validity testing. The measure requires additional development in order to determine its applicability for use in the ODDESSI research trial.

## 2. Aims

The aim of this research project is to develop and refine the DPAS (Olson et al., n.d.), for use in OD research trials in the United Kingdom (the ODDESSI program grant). The primary goal is to begin the process of psychometric formalization of a measure of OD practitioner adherence. This process will involve determining the essential components of the OD model, as defined by the OD Fidelity Criteria (Olson et al., 2014), developing a rating manual for the measure to allow it to be used by research staff throughout the project, and testing reliability and validity of the measure to determine its suitability for wider use.

## 3. Materials and methods

### 3.1. Design

This study is a mixed methods approach to the development of an OD practitioner adherence measure. Initial steps involved meetings and discussions with experts and a review of the literature to provide face validity. Content validation studies involved the use of surveys with results presented through narrative synthesis and summary statistics. To assess reliability of the measure, OD network meetings were audio-recorded, and tapes were rated by two independent researchers. Inter-rater reliability was assessed through quantitative approaches assessing variance.

### 3.2. Setting

Data for this study was drawn from the initial feasibility trial of the ODDESSI work program conducted out of University College London (UCL). This is part of the initial stages of the RCT which aims to examine the implementation of OD across different NHS trusts in England and compare outcomes to TAU. The main work for this study took place at UCL with network meeting data from North East London NHS Foundation Trust (NELFT), Kent and Medway NHS and Social Care Partnership Trust (KMPT), Barnett Enfield and Haringey NHS Trust (BEH) and Devon Partnership NHS Trust (DPT). Network meetings were recorded between September 2018 and April 2019 and rating took place between January and May 2019.

### 3.3. Therapist and patient participants

Teams established to deliver OD interventions in the above trusts participated in this research. All practitioners (psychiatrists, psychologists, social workers, nurses and peer support workers) were trained in the OD model and integrated into practicing OD teams. Clinicians had varying degrees of training in the model, some attending training in Finland to the level of being an OD trainer themselves or more trained in the United Kingdom in a one-year foundation training or three-year full training program. Practitioners obtained written consent from all service-user trial participants and their networks for meetings to be recorded and for these recordings to be used in this research.

Service users were included in the trial if they were 18 years and above and suffering from a mental health “crisis.” Mental health “crisis” included anyone who meet criteria for referral to CRTs. There is some variability in the operational definition of “crisis” across trusts and therefore additional variability in participants presenting to services in different areas due to the makeup of the population in more rural versus urban areas. Service users were excluded from the trial if they had a primary diagnosis of dementia, primary diagnosis of a learning disability, or drug and/or alcohol misuse.

A network refers to anyone closely involved in the individual service-user’s care. This includes family, friends, GPs, individual therapists, keyworkers, named nurses, members of outside agencies, etc. The service user is encouraged to identify who they would like to attend these meetings and is given the responsibility of extending these invitations on a meeting-by-meeting basis. Therefore, the make-up of each network meeting varies unpredictably in size and composition.

### 3.4. Raters

Five individuals were trained to use the measure and rate OD network meeting tapes. This included two highly trained OD practitioners who have a key role in the research trial and are involved in OD training in the United Kingdom (RR and MH), a research assistant (EW) who was involved in the research trial but does not have a background in clinical or OD work. And, finally, two trainee clinical psychologists (ML and MAM) who are not trained in the OD approach but were currently undertaking DClinPsy degrees at UCL. Raters with varying levels of background in OD were chosen in order to test whether the scale could be used by non-experts. Raters were kept blind to which practitioners were involved in the network meetings being rated, although this was not set as standard and some practitioners introduced themselves at the start of the recordings.

### 3.5. Survey participants

Individuals that attended the OD International Conference in London in 2018 were contacted *via* email to take part in an online survey. All individuals were actively researching or practicing OD and therefore had significant knowledge about the approach and various techniques applied in network meetings.

### 3.6. Procedures

#### 3.6.1. Measure development

As a starting point, collaborators (ML, RR, and MH) met to discuss the DPAS (Olson et al., n.d.), a measure developed in the United States to measure OD adherence in network meetings. The DPAS was still in development and had not undergone any validity testing. It was used as a starting point or framework from which the research team aimed to simplify the coding process and test the protocol’s reliability and validity. The first step in the process was determining the key elements of an OD network meeting using “The Key Elements of Dialogic Practice in OD: Fidelity Criteria” (Olson et al., 2014) which set out the key methods used by practitioners in OD network meetings (presented in Figure 1). These key elements were then operationalized into specific behaviors that would be witnessable to an observer. This involved debate between the collaborators (ML, RR, MH, and SP) and four drafts were produced and open to edits.

During this process researchers in the United States (Ziedonis, Small, and Larkin) were also developing an OD adherence rating manual based on the DPAS for use in their trials. This resulted in The Dialogic Practice Fidelity Rating Manual. The Dialogic Practice Fidelity Rating Manual comprised similar components to the items that were generated through the collaboration described above. It was in draft form with a more thorough description of the elements of OD than the initial DPAS with some guidance on the process of rating and scoring an OD network meeting. However, it had not undergone any validity testing and was not being regularly or widely used. Work shifted to editing and refining this measure through consultation and debate amongst collaborators to increase the ease of use and relevance to the United Kingdom trial. Refining took place across months with multiple drafts edited by collaborators with expert knowledge of the model. This was followed by the UCL rater training and analyses of reliability and validity.

#### 3.6.2. Rater training

Once the coding system was agreed upon and necessary revisions made, collaborators began a series of practice trials using the measure over a two-month period. Following familiarization with the manual, all five raters individually rating 30-min to one-hour segments of one videotaped and one audiotaped OD network meeting. Following each portion rated, raters would meet and discuss scoring and increase knowledge of OD specific techniques. During this process, each individual noted specific phrases and times within the sessions that presented confusion for discussion as a group. All raters were new to using the coding system, however two were highly trained in the model and able to answer any technical questions and aid in decision making.

Following training, the five raters listened to a complete audiotaped OD session and met to discuss the completed coding criteria. Results on the criteria were visually compared for similarities and differences amongst the raters. Differences were discussed and any conflicts addressed by group consensus. Overall, agreement was established based on these initial ratings through visual inspection of the coding sheets and average ratings across the 12 items.

#### 3.6.3. Rating

Practitioners were asked to record their network meetings with consent from the service-user and any network members present. OD sessions from different stages of treatment were included except for initial introductory sessions. There were no additional criteria that had to be met for a recording to be included in reliability analyses and, for the purposes of these analyses, it was acceptable for multiple recordings to come from the same family and same practitioners. This was because, for this study, the focus was on the utility and reliability of the measure rather than the level of adherence of the treating teams.

A 25 audio-recordings across five OD trial sites were collected for this study. Based on a literature review, this number was deemed to be acceptable and appropriate for this research (Williams et al., 2011; Pantalon et al., 2012; Gillespie, 2014; Roth, 2016). This total represented 3 audio-recordings from NELFT, 12 from KMPT, 2 from BEH, and 8 from DPT. Session length ranged from 33.02 to 115.5 min.

As this research took place in the initial pilot study stage of the RCT, no additional information was collected about service-users or practitioners other than what was on the tapes. In some sessions, introductions were made at the beginning of the recording which assisted raters in distinguishing between network member and practitioner voices. However, this was not done as standard to preserve anonymity. Therefore, it is unclear how many tapes may have been recorded by the same treating pairs or with the same network. Due to the small size of treating teams it is likely that practitioners appeared more than once on the recordings, however, there appeared to be considerable variation in service-users and networks. As tests in this study were conducted on raters rather than therapists/families this was deemed acceptable.

Initially a random number generator was used to organize the five raters into pairs and randomly allocate the tapes for independent rating. However, as the audio-recordings were collected at different time periods from December 2018 to May 2019, audio-recordings that were collected at later dates were rated purposively by available raters.

All raters except for the primary researcher were blind to their rater pairings. Raters were not given any information about scoring until after their sessions had been submitted. The primary researcher scanned score sheets for large discrepancies (for example if one rater passed a session while another failed it) and contacted raters about these sessions. This occurred on four occasions. For training purposes, raters were requested to revisit these scores, however, at no time did they see the scoresheet of the other rater. The initial scores submitted were used in the analyses.

### 3.7. Analyses

#### 3.7.1. Face/content validity

A modified Delphi technique (a method of consensus building using questionnaires) was used to gather data from respondents within their domain of expertise (Hsu and Sandford, 2007). This was done using the Qualtrics Survey Software, a free online platform for the development and data management of research surveys. Individuals with expertise in OD were contacted *via* email and sent a link to the online survey. The initial questions related to whether or not the 12 key fidelity items reflected key elements of OD practice as seen in a network meeting. Survey participants were asked to respond to this on a 5-point Likert scale from 1 (strongly disagree) to 5 (strongly agree). Respondents were then asked three further open response questions about whether they viewed these items as necessary and relevant, and whether they would make any further changes or amendments to these items. The final survey consisted of 12 Likert-response items, three qualitative feedback questions, and three respondent demographic questions.

#### 3.7.2. Inter-rater reliability

Statistical analyses were conducted using SPSS 25. Intraclass correlation coefficients (ICCs) were calculated for all pairs of coders to estimate reliability. The convention developed by Cicchetti (1994)‘s for evaluating the usefulness of ICCs was adopted for the current study and is as follows: below 0.40 = poor, 0.40 to 0.59 = fair, 0.60 to 0.74 = good, and 0.75 to 1.00 = excellent. ICC was calculated using a two-way random model with absolute agreement as per recommendations by Shrout and Fleiss (1979) for each adherence item independently as well as scale total.

#### 3.7.3. Internal consistency

Cronbach’s alpha coefficients were computed as a measure of internal consistency. A threshold of >0.70 (good) was used as a standard threshold of internal reliability (Bernstein and Nunnally, 1994). Cronbach’s alpha was selected due to the use of Likert rated items in the measure. Likert items were considered on an ordinal scale in these analyses. Reliability coefficients were inspected at the item level to determine whether or not any single items significantly impacted the overall reliability of the scale.

## 4. Results

### 4.1. Measure development

The final manual was 18 pages covering the rating process and defining the key elements of OD. The retained information and descriptions enhance understanding of meaning underlying the different elements and anchor the coding framework. The anchor points describe why a rater may give a key element a certain rating. They help to distinguish a 1 (not at acceptable level), 2 (acceptable), 3 (good), and 4 (excellent). They also clearly outline when certain decisions should be made as well as the pass/fail criteria (Forsberg et al., 2015). The four-point scale was used as it had been developed in the original manual and initial comparisons showed reliability between raters with this format. Additional anchor points on the scale would have made the rating process more complex as a greater number is likely to increase the systematic variance and redundancy in a scale (Jaju and Crask, 1999).

As part of the rating process and, in line with the definition of adherence described above, it was important to get a measure of “dose” – in this case a count of specific OD-related therapeutic techniques used within the session. In order to do this, collaborators agreed it was important to rate every “utterance” made by a practitioner. This also helped to establish the proportion or monologic versus dialogic utterances and a cut-off was established regarding the necessary proportion for a session to be true to the OD model. Collaborators created a structured table with definitions of the key elements as well as monologic items. This allowed users to tally the practitioners’ “utterances” to inform the subsequent ratings.

The 12 Likert-rated items on the scale reflect the 12 fidelity criteria (Olson et al., 2014; see Table 1), with each principle represented by one item. The first two items are structural and relate to the individuals in the room, i.e., number of practitioners and involvement of the network. The subsequent 10 items reflect the key therapeutic elements of the OD model. Final scores on the measure can range from 12 to 48. A score below 22 is considered to not be adherent (as this would represent more than two items rated as not at an acceptable level).

**Table 1:** Key elements survey results.

#	Key element	Mean	Min.	Max.	SD	Variance	Count
1	Two (or More) therapists in the team meeting	4.66	1.00	5.00	0.84	0.71	29
2	Participation of family and network	4.66	2.00	5.00	0.71	0.50	29
3	Ongoing use of open-ended questions throughout the treatment meeting as a way of linking client utterances and building dialog	4.38	3.00	5.00	0.67	0.44	29
4	Responding to clients’ utterances: This includes responsive listening, using the clients’ own words and tolerating silences in conversation	4.79	3.00	5.00	0.48	0.23	29
5	Emphasizing the present moment: Responding to immediate reactions and emotions but not interpreting or agenda setting	4.52	3.00	5.00	0.56	0.32	29
6	Eliciting multiple viewpoints: Outer and inner polyphony engaging everyone in the meeting and multiple viewpoints in an individual	4.83	4.00	5.00	0.38	0.14	29
7	Use of a relational focus in the dialog: Focus on the relational aspects of spoken stories to define relationships and elicit contextual and social information	4.24	3.00	5.00	0.68	0.46	29
8	Responding to problem discourse or behavior in a matter-of-fact style and with meaningful dialog: Seeing symptoms as “natural” responses to stressful life situations	4.41	2.00	5.00	0.77	0.59	29
9	Emphasizing the clients’ own words and stories, not symptoms: Help client find words to communicate more clearly, pay attention to one word or sub-sentences	4.69	3.00	5.00	0.53	0.28	29
10	Conversation amongst professionals (reflections) in the treatment meetings	4.48	3.00	5.00	0.72	0.53	29
11	Being transparent: Shared decision making. Disclosing Information on all discussions at the treatment meeting to all members present, sharing what clinicians do know and do not know	4.76	3.00	5.00	0.50	0.25	29
12	Tolerating uncertainty: No hasty judgments about symptoms, diagnosis or treatment, understanding and responding to the whole person in context rather than reacting to isolated behaviors	4.83	4.00	5.00	0.38	0.14	29

In order to rate these 12 items, the manual advises raters to refer to the tallies made within the utterance table and use these to inform their decision making. Simple presence or absence measures were not appropriate for use in this model because OD network meetings are led by the service-user and network and, therefore, clinicians cannot be expected to engage in all OD skills at similar levels in every meeting.

At the end of the coding sheet an overall adherence rating is taken on the basis of three general questions. In order for a session to be considered adherent a score of “Yes” has to be answered on all three yes/no questions stated below.

1. Was the proportion of dialogic statements at least two-thirds (0.67)?
2. Were at least 8 of the 10 fidelity items in Section B at the level of “Acceptable” or higher?
3. Were there fewer than two instances of patronizing or disrespectful statements?

### 4.2. Validity

#### 4.2.1. Face validity

A large extent of face validity of the measure was established through the parallel development process in both the United States and United Kingdom. The measure was also based on the theoretical concepts outlined by Olson et al. (2014) which provides a strong theoretical grounding based on international expert opinion.

#### 4.2.2. Content validity

Twenty-nine individual responses were received *via* the Qualtrics Survey Software. Survey participants varied in levels of training/experience from expert >5-years (*N* = 12), advanced 2-5-years (*N* = 11) and beginner <2-years (*N* = 6). All individuals were actively researching or practicing OD and therefore all had large amounts of knowledge in the area. Nine participants were primarily involved in OD research, 9 in OD practice and 11 involved in both research and practice. Participants represented an international sample (Australia = 4; Belgium = 1; Finland = 5; France = 1; Germany = 2; Italy = 1; Japan = 1; Lithuania = 2; Norway = 1; Netherlands = 2; United Kingdom = 7; United States = 1; Unknown = 1).

Results from question one of the survey are presented below in Table 1. Participants were asked “To what extent do the following items represent key elements of OD Practice as would be seen in a network meeting?” and respondent on a Likert scale as described in the methods. Mean ratings for each element was above 4.0 representing agreement for all 12 items.

**Figure 2:**
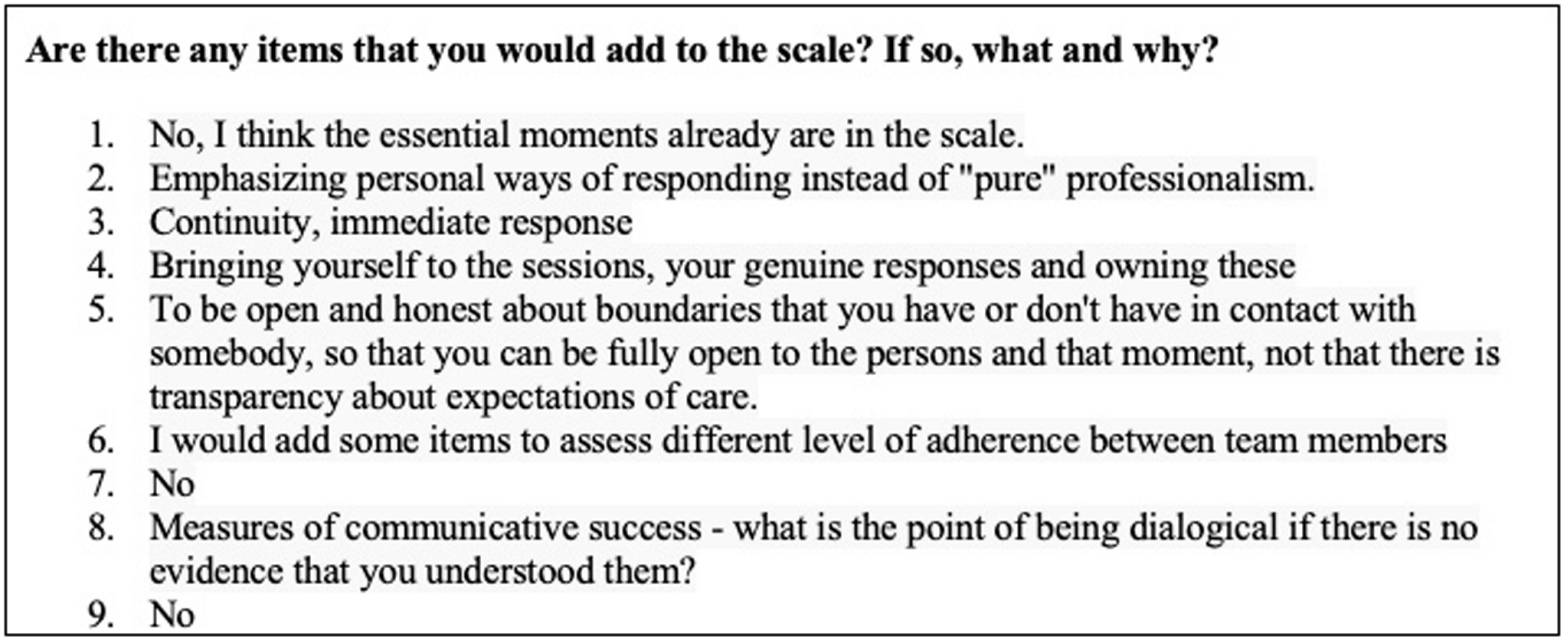
Items to add to the scale.

**Figure 3:**
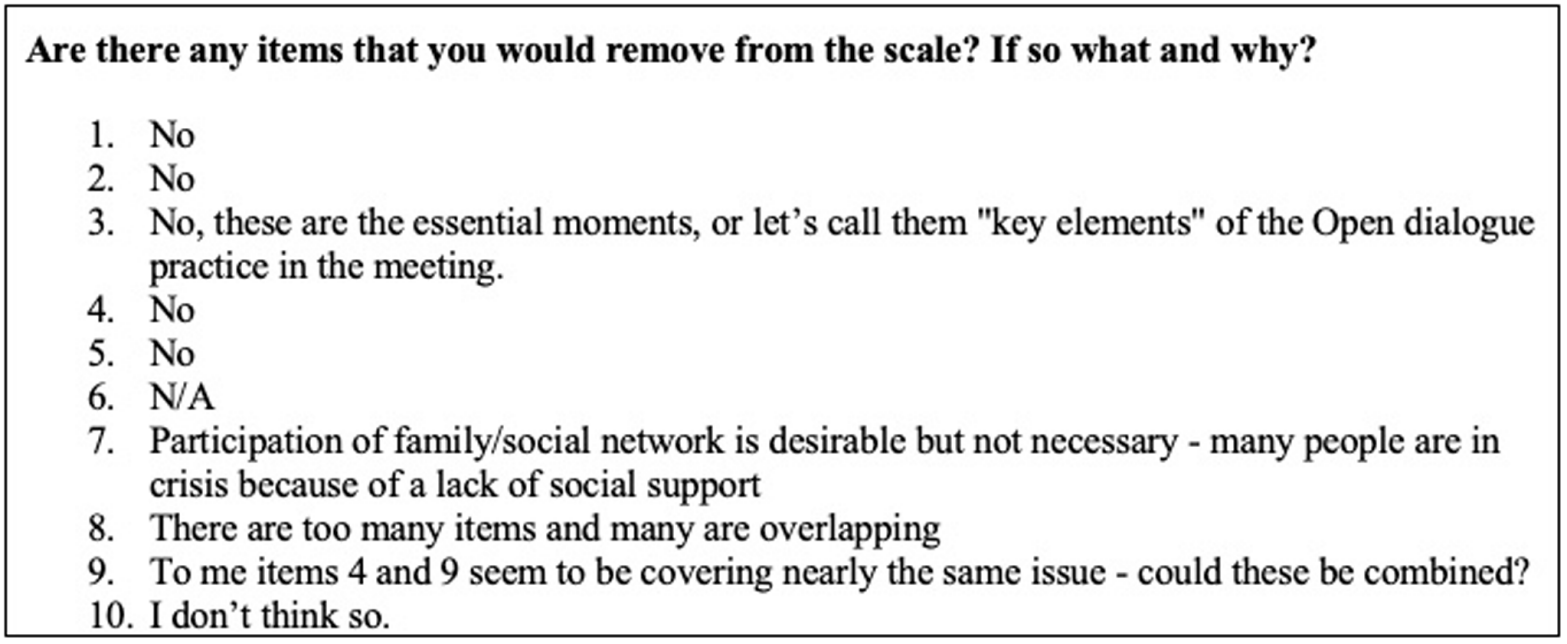
Items to remove from the scale.

**Figure 4:**
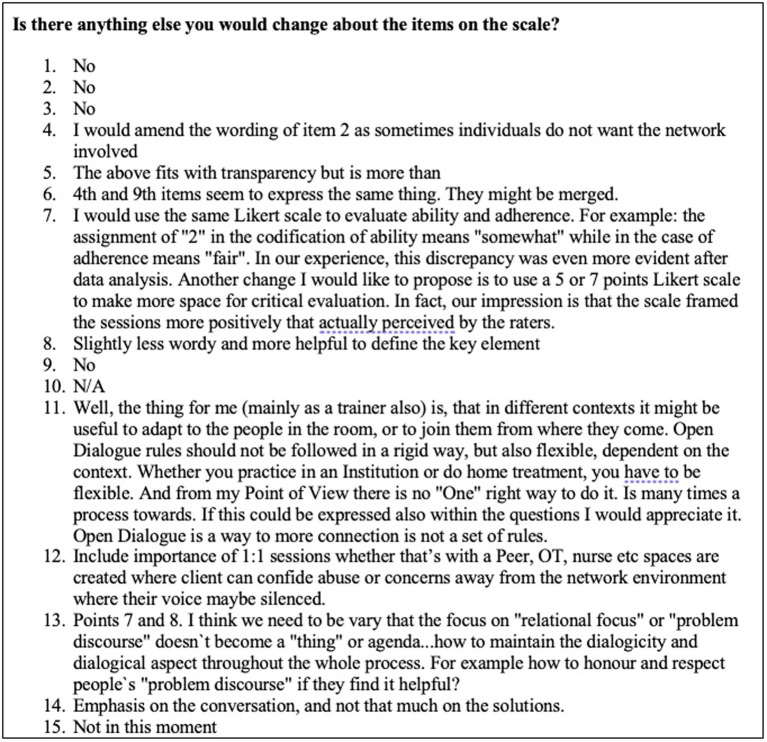
Changes to the scale.

Participants were also asked the following open response questions: (1) What you would add to the scale? (2) What would you remove from the scale? and (3) Is there anything you would change? These questions received variable responses and are presented below (see Figures 2–4).

Overall 6 of 29 survey respondents suggested items that they would add to the scale (see Figure 2). Many of these responses (i.e., numbers 1, 4, and 5) related to openness of response and genuineness of clinicians. Response 3 refers to an aspect of OD team structure better captured in a fidelity measure. And, response 6 advises different measures of adherence for each clinician to capture cases when one clinician may be more or less adherent than the other.

Only three of 29 respondents suggested removing any items from the scale (see Figure 3). Two of these suggested potential overlaps between items, e.g., items 4 and 9. The other response suggested decreasing the relevance of social network participation within the measure.

The final question about changes to the scale received the most responses, however, many of these responses advocated keeping the present measure (see Figure 4). One response (number 7) recommended changes in scaling used. Two (4 and 6) echoed changes advised in Figure 3 to item 2 and combining items 4 and 9. Response 12 refers to additional interventions outside of network meetings which is outside the remit of this measure. Many responses reflect the importance of clinicians being flexible and not applying specific techniques unless it fits with the nature of the current network meeting.

### 4.3. Scale output

Means and standard deviations for each item were computed (see Table 2). Average over all score was 33.16 out of 44 (*N* = 50) showing that, overall, sites were adherent as rated on the measure. Average scores on each item ranged from adherent to good with the lowest average score on item 7 (relational focus) and the highest average score on item 4 (responsive listening).

**Table 2:** Inter-rater reliability and adherence descriptors.

Item	Description (*N* = 25)	Mean Score	SD.	ICC
*Total*		*33.16*	*6.011*	*0.906*
Avg.				
1	Two (or More) therapists in the team meeting	3.06	0.682	0.612
2	Participation of family and network	2.64	0.898	0.792
3	Ongoing use of open-ended questions throughout the treatment meeting as a way of linking client utterances and building dialog	2.62	0.878	0.675
4	Responding to clients’ utterances: This includes responsive listening, using the clients’ own words and tolerating silences in conversation	3.16	0.889	0.573
5	Emphasizing the present moment: Responding to immediate reactions and emotions but not interpreting or agenda setting	2.68	0.891	0.824
6	Eliciting multiple viewpoints: Outer and inner polyphony engaging everyone in the meeting and multiple viewpoints in an individual	2.52	0.839	0.707
7	Use of a relational focus in the dialog: Focus on the relational aspects of spoken stories to define relationships and elicit contextual and social information	2.24	0.847	0.669
8	Responding to problem discourse or behavior in a matter-of-fact style and with meaningful dialog: Seeing symptoms as “natural” responses to stressful life situations	2.84	0.766	0.734
9	Emphasizing the clients’ own words and stories, not symptoms: Help client find words to communicate more clearly, pay attention to one word or sub-sentences	3.04	0.781	0.704
10	Conversation amongst professionals (reflections) in the treatment meetings	2.58	0.835	0.727
11	Being transparent: Shared decision making. disclosing information on all discussions at the treatment meeting to all members present, sharing what clinicians do know and do not know	2.72	0.757	0.678
12	Tolerating uncertainty: No hasty judgments about symptoms, diagnosis or treatment, understanding and responding to the whole person in context rather than reacting to isolated behaviors	2.90	0.735	0.625

Italicized values are the total adherence scores.

### 4.4. Reliability

#### 4.4.1. Inter-rater reliability

Inter-rater reliability for the total score was excellent. The average measure ICC was 0.906 with a 95% confidence interval from 0.785 to 0.958 [*F*(24,24) = 10.254, *p* < 0.001]. ICCs for each discrete item ranged from fair to excellent with most items in the good (*N* = 6) and excellent (*N* = 5) range. The one item which fell below this was item 4 (responsive listening; ICC = 0.573).

#### 4.4.2. Internal consistency

Calculation of Cronbach’s alpha for the 12 items was highly reliable (α = 0.848). There was no item that could be removed from the scale to substantially increase internal consistency and all items had high item total correlations.

## 5. Discussion

The aim of this study was to develop and psychometrically formalize a measure of OD practitioner adherence for use in the United Kingdom-based ODDESSI RCT. The initial goal of this study was to develop and refine the DPAS (in development) which had previously been developed to rate dialogic practices within network meetings. However, as the study progressed a new measure was developed, and this is presented here. Validity of the new OD Adherence Scale has been established and internal consistency statistics report that the scale is reliable meeting the initial aims of this research project.

This is the first study to analyze the psychometric properties of the OD Adherence Scale and the results from the application of the measure provided initial adherence data which was required by NIHR in the feasibility stage of this trial. Using the scale, it was found that therapists practicing OD in the participating NHS trusts were adherent in delivery of core OD interventions. Average scores were in the adherent to good range overall and for individual items. This was true across trusts who served different populations and therefore had variability in the presentations seen within their services. It also held true with different network types and compositions.

Psychometric properties of the scale suggest that this tool may be useful in assessing adherence in OD. Modified Delphi results show that OD experts and new practitioners agree that the scale represents the key elements of the OD theoretical model. There were minimal changes suggested for the scale and many of these related to elements that would be better covered in a fidelity scale or items that are not easily operationalized for an observer rated tool. For example, individual support offered to the service user outside of network meetings would not be something observable in network meetings and would require additional interviews with service users and staff which is outside of the remit of this measure.

The use of different levels of adherence rating (adherent, good and excellent) allows the rater to make judgments about how the intervention was received by the network, whether it was appropriate, and whether or not it worked well in the context. The use of these additional rating points allows for flexibility in the sessions and addresses concerns about the rigidity of the scale described in the results. For example, neither the manual nor the measure specifies the number of occurrences of a technique for reliability. Therefore, a technique can still be rated as excellent despite occurring infrequently while another may be rated as poor in spite of occurring many times during a session. This is important for a therapeutic model such as OD with a focus on unique and flexible responses to each network in each session.

Inter-rater reliability for the overall adherence score was excellent (Shrout and Fleiss, 1979). High inter-rater reliability indicates that two randomly selected raters reliably discriminated clinician’s use of and competence in different therapeutic techniques (Haddock et al., 2001) and the excellent overall score suggests that the OD Adherence Scale is a highly reliable measure. ICC ranged from fair to excellent across the items with the lowest score for item 4 responsive listening. Systematic differences between raters would likely be due to differing levels of experience both in clinical work and in OD practice. However, agreement was high for the overall score and 11 of the 12 items suggesting that training completed as part of the measure development process was sufficient, even for those with less experience with the OD model. It also shows that the measure is accessible to those with less exposure to OD and general clinical work increasing its utility in different contexts.

The measure also demonstrated a high level of internal consistency (as reported by Cronbach’s alpha) suggesting that it is a reliable measure of the intervention and that competent delivery of one individual therapeutic technique is related to competent delivery of the others (Forsberg et al., 2015). However, Cronbach’s alpha is not a measure of how many constructs were measured by the scale. Additional data along with further investigation is needed to explore whether OD adherence can be efficiently rated as one global dimension.

### 5.1. Limitations

An important limitation of this study is the limited sample size. Significant resource is required to rate full length therapy sessions (Perepletchikova et al., 2009) and this is particularly true of OD sessions which can range from 40-min to two-hours in length. Ideally, each of the five individual raters would have independently rated each OD tape but such resource was not available for this study. Low sample size may have contributed to variability in inter-rater reliability and internal consistency, which may have been improved with a larger sample (Shrout and Fleiss, 1979; Forsberg et al., 2015).

Additionally, there was a large time delay in receiving audio-recordings from sites which impacted the randomization process. Raters were initially randomized into pairs and to tapes but this process became purposive nearing the end of the study due to time constraints. Randomization of recordings was conducted by session, not by participant or site, therefore we had different numbers of sessions per site and there may have been some sampling bias by clinicians. As this research took place in the pilot stage of the trial, we did not collect identifying information about service users or practitioners which did not allow us to determine the impact of who was recorded on reliability outcomes. This information will be collected at later stages in the trial.

### 5.2. Strengths and future directions

The OD Adherence Scale is the first attempt to identify and operationalize the key elements of an OD network meeting. This study provides evidence of a consensus on the key elements of OD network meetings and dialogic practice. A strength of this research is having a varied and international team of researchers involved in the development of the measure. The parallel development processes in the United Kingdom and United States provides additional evidence of the validity of the measure. The scale presented here is an initial attempt at rating practitioner adherence in these meetings. It provides encouraging evidence that this can be done with good validity and reliability and can be completed by a range of raters with different levels of clinical experience. The scale is easy to use and does not take much longer than a network meeting to complete. It will be an important addition to OD implementation research which must report on whether OD theoretical techniques are being used adequately in practice.

This study also provides initial psychometric information as the foundation for future research and additional validation of the OD Adherence Scale. It is recommended that, as more data is collected using the measure, further analyses be performed such as those listed in the above limitations. This will improve our understanding of the measures psychometric properties providing additional evidence for or against its utility moving forward.

The manual produced as part of this research has now replaced those in development. It is being used to train raters in the United Kingdom and internationally as countries implement OD into their mental health care systems.

## 6. Conclusion

Perepletchikova and Kazdin (2005) propose that, in order to achieve greater scientific validity, studies looking at the relationship between fidelity and outcome should investigate empirically supported treatments, use validated fidelity measures rated by non-participant judges, and control for third variable influences. This study provides the initial element of this process for the ODDESSI program by providing psychometric information on the OD Adherence Scale.

Monitoring adherence is necessary for assessing whether participants or service users are receiving the appropriate evidence-based treatment and to identify when and how this goes wrong (Walton, 2018). It has implications for providers and wider systems and leaves us with ethical questions about how we should deliver treatment. While “perfect or near-perfect” implementation is unrealistic (Dulak and DuPree, 2008) it remains important to measure fidelity of delivery and to report on it transparently and clearly in order to translate interventions into real world settings (Walton, 2018).

Knowledge of fidelity and adherence in OD needs further development. This study is an important first step in the OD Adherence Scale’s evaluation and validation. However, the initial results presented here provide a promising foundation for the OD Adherence Scale’s utility within OD research projects.

## Data availability statement

The raw data supporting the conclusions of this article will be made available by the authors, without undue reservation.

## Author contributions

ML wrote the text of this article and completed all surveys and statistical analyses. DZ provided a measure that he had begun development on (as described in the text). ML, RR, MA, and MH met to determine key features of OD and developed iterations of the original measure. ML, RR, MA, MH, and EW trained together to rate tapes and acted as raters and were involved in meetings to discuss and determine reliability. SP provided the idea for this research project as well as mentorship throughout and acted as an editor of this text and provided ideas for analyses. All authors contributed to the article and approved the submitted version.

## Funding

This study was funded by Open Dialogue: Development and Evaluation of a Social Network Intervention for Severe Mental IIlness (ODDESSI) (Grant no. RP-PG-0615-20021).

## Conflict of interest

The authors declare that the research was conducted in the absence of any commercial or financial relationships that could be construed as a potential conflict of interest.

The reviewer TB declared a past co-authorship with the author MH to the handling editor.

## Publisher’s note

All claims expressed in this article are solely those of the authors and do not necessarily represent those of their affiliated organizations, or those of the publisher, the editors and the reviewers. Any product that may be evaluated in this article, or claim that may be made by its manufacturer, is not guaranteed or endorsed by the publisher.

## Footnotes

^1^ https://www.ucl.ac.uk/core-study
